# Evolution of isoprenyl diphosphate synthase-like terpene synthases in fungi

**DOI:** 10.1038/s41598-020-71219-z

**Published:** 2020-09-10

**Authors:** Guo Wei, Franziska Eberl, Xinlu Chen, Chi Zhang, Sybille B. Unsicker, Tobias G. Köllner, Jonathan Gershenzon, Feng Chen

**Affiliations:** 1 Department of Plant Sciences, University of Tennessee, Knoxville, TN 37996 USA; 2 Department of Biochemistry, Max Planck Institute for Chemical Ecology, Hans-Knöll Str. 8, 07745 Jena, Germany

**Keywords:** Biochemistry, Molecular biology

## Abstract

Terpene synthases (TPSs) and *trans*-isoprenyl diphosphate synthases (IDSs) are among the core enzymes for creating the enormous diversity of terpenoids. Despite having no sequence homology, TPSs and IDSs share a conserved “α terpenoid synthase fold” and a trinuclear metal cluster for catalysis, implying a common ancestry with TPSs hypothesized to evolve from IDSs anciently. Here we report on the identification and functional characterization of novel IDS-like TPSs (ILTPSs) in fungi that evolved from IDS relatively recently, indicating recurrent evolution of TPSs from IDSs. Through large-scale bioinformatic analyses of fungal IDSs, putative ILTPSs that belong to the geranylgeranyl diphosphate synthase (GGDPS) family of IDSs were identified in three species of *Melampsora*. Among the GGDPS family of the two *Melampsora* species experimentally characterized, one enzyme was verified to be *bona fide* GGDPS and all others were demonstrated to function as TPSs. *Melampsora* ILTPSs displayed kinetic parameters similar to those of classic TPSs. Key residues underlying the determination of GGDPS versus ILTPS activity and functional divergence of ILTPSs were identified. Phylogenetic analysis implies a recent origination of these *ILTPSs* from a *GGDPS* progenitor in fungi, after the split of *Melampsora* from other genera within the class of Pucciniomycetes. For the poplar leaf rust fungus *Melampsora larici-populina*, the transcripts of its *ILTPS* genes were detected in infected poplar leaves, suggesting possible involvement of these recently evolved *ILTPS* genes in the infection process. This study reveals the recurrent evolution of *TPSs* from *IDSs* since their ancient occurrence and points to the possibility of a wide distribution of *ILTPS* genes in three domains of life.

## Introduction

Terpenoids constitute the largest class of metabolites made by living organisms with > 80,000 structures recorded so far^1^. Yet only a small portion of the described terpenoids is known to be essential for the basic physiology of the producing organisms. Examples include steroids as membrane components, carotenoids as photosynthetic pigments, and various types of terpenoid-derived hormones in plants and animals. The majority of terpenoids are classified as secondary metabolites. These include substances of enormous skeletal diversity that are not believed to be essential for cellular and physiological processes, but rather for interactions with other organisms^2,3^.

Much has been learned about the mechanisms that create the huge diversity of terpenoids ever since the days of Leopold Ruzicka and his ‘biogenetic isoprene rules’^4,5^. Two of the major classes of enzymes that underlie terpenoid diversity are the isoprenyl diphosphate synthase (IDS) and terpene synthase (TPS). Isoprenyl diphosphate synthases catalyze the formation of the ubiquitous acyclic isoprenoid intermediates of various chain lengths from isopentenyl diphosphate (IDP) and dimethylallyl diphosphate (DMADP) and thus control the size of the final products. The products include geranyl diphosphate (GDP, C_10_), farnesyl diphosphate (FDP, C_15_), and geranylgeranyl diphosphate (GGDP, C_20_)^6^. There are two distinct types of IDSs: those synthesizing prenyl diphosphate units with *E*-double bonds and those synthesizing prenyl diphosphate units with *Z*-double bonds^7^. The former type, known as *trans*-IDSs, is involved in the formation of most low molecular weight terpenoids.

The other major enzyme class controlling terpenoid diversity, the TPSs, converts GDP, FDP, and GGDP to monoterpene, sesquiterpene, and diterpene skeletons, respectively^8,9^. Various types of *TPS* genes have been described^1,10^, such as typical plant *TPS* genes, microbial *TPS*-like (*MTPSL*) genes, and *TPS* genes from various microorganisms. Here they are collectively termed classic *TPS* genes. Despite having no significant sequence homology, TPSs and IDSs share a conserved “terpenoid synthase fold”, also known as the “α” domain^11,12^, implying a shared evolutionary origin. Moreover, at the level of reaction mechanism both IDSs and TPSs employ a conserved trinuclear metal cluster for catalysis, which also implies a common ancestry of the two types of enzymes^13^. Because the production of prenyl diphosphates by IDSs is central to the terpene biosynthetic pathway and supplies substrates for TPSs, the origin of IDSs should have predated that of TPSs. Thus, one major evolutionary scenario is that TPSs evolved from IDSs^12^. Lack of significant sequence homology between TPSs and IDSs and their apparent divergence before the split of prokaryotes and eukaryotes suggest that this divergence occurred anciently.

One central hypothesis of this present work is that the evolution of TPSs from IDSs is a recurrent process that has happened at different times in evolution since their ancient occurrence. While IDSs are ubiquitous, *TPS* genes have a patchy distribution, occurring only in plants, fungi, bacteria, and social amoeba^9,14–16^. Some organisms do not contain *TPS* genes but are known to use terpenoids for adaptive functions, raising the question whether they are synthesized by novel, non-classic *TPS* genes. Another direct evidence to support this hypothesis comes from the studies on terpenoid pheromones in a number of insect species, in which the precursors of terpenoid pheromones were demonstrated to be synthesized by IDS-like enzymes belonging to the farnesyl diphosphate synthase (FDPS) family^17–20^. It is notable that insects do not contain classic *TPS* genes. To test our hypothesis that the evolution of *TPS* genes from *IDS* genes is a recurrent progress and has occurred independently in various lineages, we here investigate fungi. Like insects, fungi are a species-rich group of organisms^21,22^ of which many produce terpenoids in high abundance^23^. Since fungi are known to contain several types of classic *TPS* genes of ancient phylogenetic origin^14^, it would be particularly interesting to determine if TPSs in this group have also evolved more recently from IDSs.

## Results

### Identification of putative IDS-like TPSs in fungi that belong to the GGDPS family

**Figure 1:**
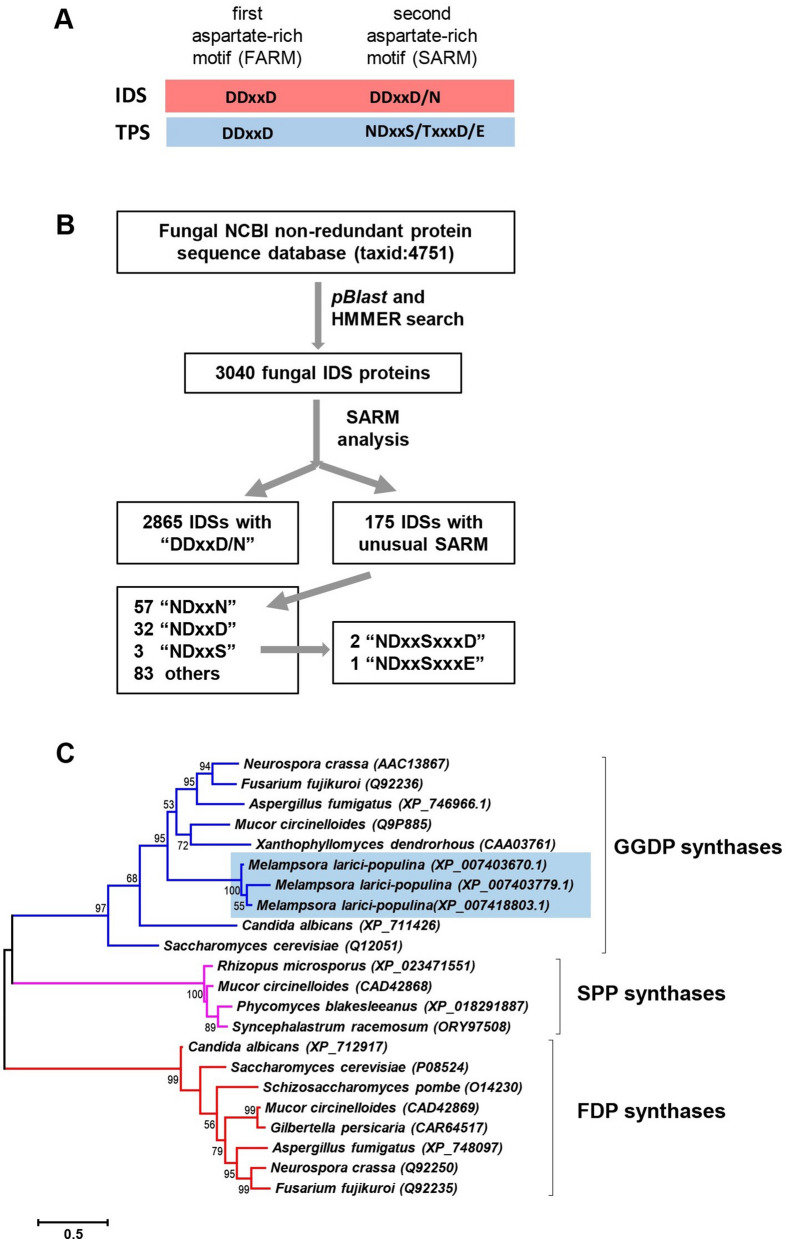
Schematic illustration and evolutionary analysis of candidate IDS like TPSs in fungi. (**A**) The canonical ‘DDx_2–4_D’ and “NSD” triad present in both IDSs and TPSs, respectively. (**B**) Sequence analysis of fungal IDSs and identification of ones with specific second aspartate rich motif (SARM). The fungal IDS protein sequences were obtained by searching the fungal non-redundant database (taxid:4751) from NCBI followed by HMMER search. SARM in all candidate protein sequences were aligned and analyzed to identify the conserved and unusual motif in these sequences. (**C**) Phylogenetic analysis of three candidate IDS type TPSs with protein sequences of known FDPSs, GGDPSs, and SPPSs. All sequences were downloaded from the NCBI database and their accession numbers are indicated on the tree.

The trinuclear metal cluster in both IDSs and class I TPSs are coordinated by metal-binding motifs^13^. There are two general types: the “aspartate-rich” motif in the form of ‘DDx_2_-_4_D’ and the “NSD” triad most typically in the form of ‘N/DDxxS/TxxxD/E’. IDSs generally contain two “aspartate-rich” motifs, which are also known as FARM (first aspartate rich motif) and SARM (second aspartate rich motif), respectively. For SARM, one variation is ‘DDxxN’ observed in some geranylgeranyl diphosphate synthases (GGDPSs), such as the human GGDPS^24^. In contrast, TPSs from bacteria, fungi, and plants generally possess one “aspartate-rich” motif and one “NSD” triad (Fig. 1A). We set to use this well-observed distinction between the SARM of IDSs and of “NSD” triad of TPSs^12^ as a criterion to identify putative IDS-like *TPS* genes in fungi. A scheme illustrating our experimental approach and the data acquired at various stages is shown in Fig. 1B. From National Center for Biotechnology Information (NCBI) non-redundant fungal protein sequence database (taxid:4751), a total of 3,040 fungal IDS proteins with a length of 200–500 amino acids was identified using BLASTP and HMMER search. Among them, 2,865 proteins contain the highly conserved “DDxxD/N” SARM. Within the remaining 175 IDSs, 92 proteins contain “NDxxx” SARM. Among the 92 IDSs, three contain “NDxxS” SARM with two of them being “NDxxSxxxD” and one being “NDxxSxxxE”. These three represented putative IDS-like TPSs (ILTPSs) based on the criterion we initially set. Interestingly, all these three putative ILTPSs are from the same fungus *Melampsora larici-populina,* the poplar leaf rust fungus. Next, we performed phylogenetic analysis of the three putative ILTPSs with selected IDSs proteins of known types: FDPS, GGDPS, and solanesyl diphosphate synthase (SPPS). The three putative ILTPSs are in the same clade with known GGDPSs (Fig. 1C). Therefore, we concluded that the putative ILTPSs from *M. larici-populina* belong to the GGDPS family.

### GGDPS family of three species of *Melampsora* containing putative IDS-like TPSs

Because the genome of *M. larici-populina* has been fully sequenced^25^, we thoroughly searched its genome sequence for putative *GGDPS* genes and could identify four. In addition to the three putative ILTPSs that we have identified through the large-scale bioinformatic analysis (Fig. 1), a fourth GGDPS containing a canonical “DDxxN” SARM was identified (Table 1). The three ILTPSs were designated MlpILTPS1-3 and the GGDPS gene was designated MlpGGDPS. Within the genus *Melampsora*, besides *M. larici-populina*, the genomes of two other species have also been fully sequenced: *Melampsora lini*^26^ and *Melampsora allii­populina* (https://genome.jgi.doe.gov/portal/). For comparison, we also searched the genome sequences of *M. lini* and *M. allii-populina* for putative *GGDPS* genes. *M. lini* also contains four putative genes annotated as members of the *GGDPS* gene family (Table 1). Two of them contain a “DDxxN” SARM. Our later biochemical characterization showed that one of them encodes GGDPS and thus was designated MliGGDPS. The other one gene was demonstrated to encode a TPS and thus was named MliILTPS1. The remaining two proteins contain the “NDxxSxxD/E” motif and were designated MliILTPS2 and MliILTPS3. In contrast, five putative genes in the genome of *M. allii-populina* were annotated to belong to the GGDPS family (Table 1). Three of them contain a ‘DDxxN’ SARM. Based on their sequence relatedness to those in *M. larici-populina* and *M. lini*, one of them was designated MapGGDPS and the rest MapILTPS1-4. Notably, MapILTPS1 and MapILTPS2 are 100% identical. Both MapILTPS3 and MapILTPS4 contain the ‘NDxxSxxxD’ motif. Sequence comparisons between GGDPS and putative IDS-like TPSs from the three species of *Melampsora* revealed that MlpGGDPS, MliGGDPS, and MapGGDPS are longer than ILTPSs due to an extended N-terminus of approximately 40 amino acids (Fig. S1).

**Table 1:** The family of GGDPS from three species of *Melampsora.*

Species	Gene ID	Gene name	SARM
*M. larici-populina*	Mellp1.estExt_Genewise1Plus.C_80034	MlpGGDPS	DDLLNLSSV
	Mellp1.EuGene.00010468	MlpILTPS1	NDLLSLSAD
	estExt_fgenesh1_pm.C_LG_03_t10275	MlpILTPS2	NDLLSLSRE
	Mellp1.e_gw1.1.335.1	MlpILTPS3	NDLLSLSRD
*M. lini*	MELLI_sc_2159.2	MliGGDPS	DDLLNLSSV
	MELLI_sc_1240.3	MliILTPS1	DDLLNLSPA
	MELLI_sc_808.4	MliILTPS2	NDLLSLSPD
	MELLI_sc_1329.5	MliILTPS3	NDISSLSPD
*M. allii-populina*	estExt_fgenesh1_pm.C_1110022	MapGGDPS	DDLLNLSSV
	e_gw1.2194.14.1	MapILTPS1	DDLLNLSPA
	e_gw1.1815.21.1	MapILTPS2	DDLLNLSPA
	MIX51276_5_47	MapILTPS3	NDLLSLSPD
	fgenesh1_pm.177_#_11	MapILTPS4	NDISSLSPD

SARM, second aspartate rich-motif.

### ILTPSs lack coupling activity

**Figure 2:**
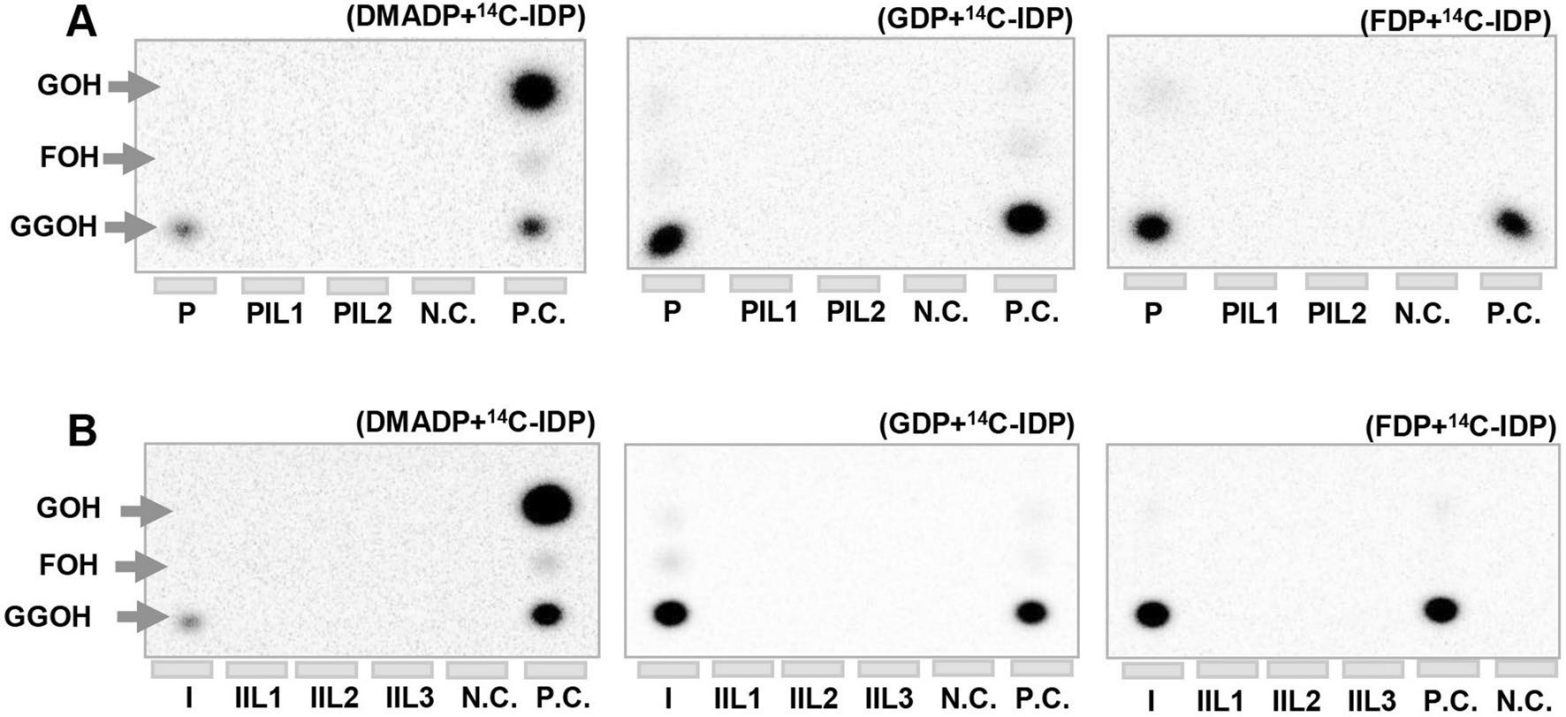
In vitro IDS enzymatic assays of the GGDPSs and ILTPSs. (**A**) Assays of MlpGGDPS (P), MlpILTPS1 (PIL1), MlpILTPS2 (PIL2) from *Melampsora larici-populina* using DMADP, GDP, and FDP together with [1-^14^C]-IDP as substrates. (**B**) Assays of MliGGDPS (I), MliILTPS1 (IIL1), MliILTPS2 (IIL2) and MliILTPS3 (IIL3) from *Melampsora lini* using DMADP, GDP, and FDP together with [1-^14^C]-IDP as substrates. In both (**A**) and (**B**), the reaction products were separated via thin-layer chromatography (TLC). CsaLSU/CsaSSU I from cucumber was used as a positive control (P.C.). Boiled CsaLSU/CsaSSU I was used as a negative control (N.C.). *GOH* geraniol, *FOH* farnesol, *GGOH* geranylgeraniol. The final products were determined by phosphor-imaging and displayed with ImageJ software. Full-length blots are presented in Fig. S3.

Despite containing an “NSD” triad, all putative ILTPSs are highly homologous to known GGDPSs (Fig. S1), raising the question of whether they retain the IDS activity. To this end, we selected *M. larici-populina* and *M. lini* for further experimental studies. From mixed RNAs of poplar leaves infested with *M. larici-populina*, we performed reverse transcription PCR (RT-PCR) to amplify the full-length cDNAs of MlpGGDPS and MlpILTPSs. Except for MliILTPS3, which was not amplified, full-length cDNAs of MlpGGDPS, MlpILTPS1, and MlpILTPS2 were amplified and cloned into a protein expression vector pET32-a. For *MliGGDPS* and *MliILTPS* genes from *M. lini*, their respective full-length cDNAs were synthesized and cloned into pET32-a. Next, all seven cDNAs in pET32-a were expressed in *Escherichia coli* to produce recombinant proteins. Purified proteins (Fig. S2) were tested for IDS activities using [1-^14^C]-IDP and three allylic substrates DMADP, GDP, and FDP. The radiolabeled products were separated by reversed-phase thin-layer chromatography (TLC) and identified by comparison to standards. A plasmid consisting of both a heteromeric GGDPS and an inactive small subunit (type I) (CsaLSU/CsaSSU I) of cucumber (*Cucumis sativus*), which together produce predominantly GDP and GGDP, was used as a positive control^27^. For *M. larici-populina*, MlpGGDPS catalyzed the formation of GGDP using either DMADP, GDP, or FDP with [1-^14^C]-IDP as co-substrate. In contrast, neither MlpILTPS1 nor MlpILTPS2 was active with any of the three allylic substrates tested (Fig. 2A). For *M. lini*, MliGGDPS was active with DMADP, GDP, and FDP to produce GGDP. Like MlpILTPSs, all three MliILTPS proteins failed to produce prenyl diphosphate products with any of the three allylic substrates (Fig. 2B).

### *ILTPS* genes encode active terpene synthases

With MlpGGDPS and MliGGDPS demonstrated to be *bona fide* GGDPS and all the other five ILTPSs from *M. larici-populina* and *M. lini* proved to lack IDS activity (Fig. 2), next we performed terpene synthase enzyme assays to determine whether these five ILTPSs indeed function as terpene synthases. The five recombinant ILTPS proteins were tested with three common substrates of classic TPSs: GDP, FDP, and GGDP. MlpGGDPS and MliGGDPS were also analyzed as negative controls.

**Figure 3:**
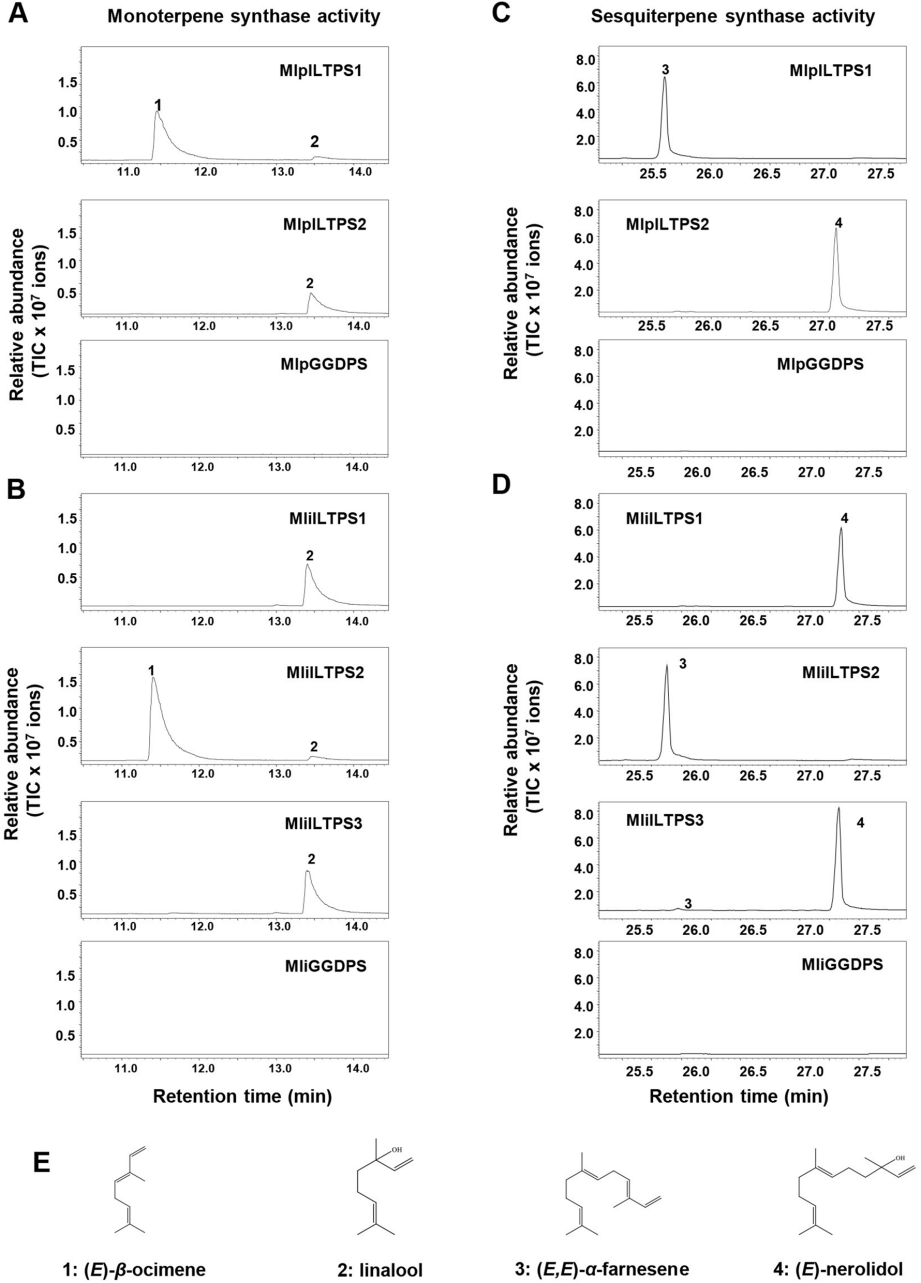
GC chromatograms of the terpene synthase enzyme assays. (**A**) In vitro monoterpene products formed from recombinant MlpGGDPS and MlpILTPSs using GDP as substrate. (**B**) In vitro monoterpene products formed from recombinant MliGGDPSs and MliILTPSs using GDP as substrate. (**C**) In vitro sesquiterpene products formed from recombinant MlpGGDPS and MlpILTPSs using FDP as substrate. (**D**) In vitro sesquiterpene products formed from recombinant MliGGDPSs and MliILTPSs using FDP as substrate. All products were analyzed by gas chromatography-mass spectrometry (GC–MS), and the total ion chromatograms (TIC) are shown. 1, (*E*)-β-ocimene; 2, linalool; 3, (*E,E*)-α-farnesene; 4, (*E*)-nerolidol. (**E**) Structures of the two monoterpene and two sesquiterpene products. Linalool and (*E*)-nerolidol were identifed using authentic standards (Fig. S4). (*E*)-β-ocimene and (*E,E*)-α-farnesene were identified based on comparison of their respective Kovats retention index (Table S2).

When GDP was provided as a substrate, MlpILTPS1 produced (*E*)-*β*-ocimene as a major product and linalool as a minor product. In contrast, MlpILTPS2 produced only linalool (Fig. 3A). When MlpGGDPS was tested at same conditions, no monoterpene product was detected (Fig. 3A). For *M. lini*, both MliILTPS1 and MliILTPS3 converted GDP to linalool, whereas MlpILTPS2 produced (*E*)-*β*-ocimene as the major monoterpene product (Fig. 3B). MliGGDPS did not produce monoterpenes from GDP (Fig. 3B).

In the sesquiterpene synthase activity assays using FDP as substrate, MlpILTPS1 produced (*E,E*)-*α*-farnesene and MlpILTPS2 catalyzed the formation of (*E*)-nerolidol (Fig. 3C). When MlpGGDPS was tested with FDP at same conditions, no sesquiterpene was detected (Fig. 3C). For *M. lini*, both MliILTPS1 and MliILTPS3 converted FDP to (*E*)-nerolidol, whereas MliILTPS2 produced (*E,E*)-*α*-farnesene as the major sesquiterpene product (Fig. 3D). Similar to MlpGGDPS, MliGGDPS did not produce any sesquiterpene from FDP (Fig. 3D). GGDP was also tested as substrate for all seven proteins. Only MlpILTPS1 and MliILTPS2 were active with GGDP. They both produced (*E,E,E*)-α-springene from GGDP (Fig. S5). It is notable that all the terpene products detected in our study are acyclic compounds (Fig. 3E).

To further exclude the possibility that the acyclic terpenes detected from in vitro enzyme assays with ILTPSs were the result of non-enzymatic solvolysis of prenyl diphosphates, we analyzed these enzymes using an in vivo approach that relies on the internal pool of FDP in *E. coli*^28^. The production of sesquiterpene products by ILTPSs (Fig. S6) was consistent with that of the in vitro approach (Fig. 3). Similarly, no terpenes were detected in the headspace of cultures expressing MlpGGDPS or MliGGDPS (Fig. S6). To gather information about the relevance of the original homoallylic diphosphate binding site on the catalytic function of ILTPSs, MliILTPS2 was selected for terpene synthase enzyme assays using FDP as substrate in the presence of IDP. The product profile of MliILTPS2 was unaffected (Fig. S7).

Next, MliILTPS2 and MliILTPS3 were selected as examples to determine their kinetic properties as terpene synthases. In this experiment, FDP was used as substrate. Under steady state conditions, the apparent *K*_m_ values of MliILTPS2 and MliILTPS3 using FDP as substrate were determined to be 1.9 ± 0.3 µM and 1.6 ± 0.2 µM, respectively. The *k*_cat_ values of MliILTPS2 and MliILTPS3 were 0.01 ± 0.001 s^−1^ and 0.05 ± 0.004 s^−1^, respectively. Such kinetic parameters of MliILTPS2 and MliILTPS3 are comparable to those of classic TPSs^29^, suggesting that MliILTPS2 and MliILTPS3 function in almost the same way as classic TPSs.

### Key amino acids for converting GGDPS to TPS and for functional divergence of ILTPSs

**Figure 4:**
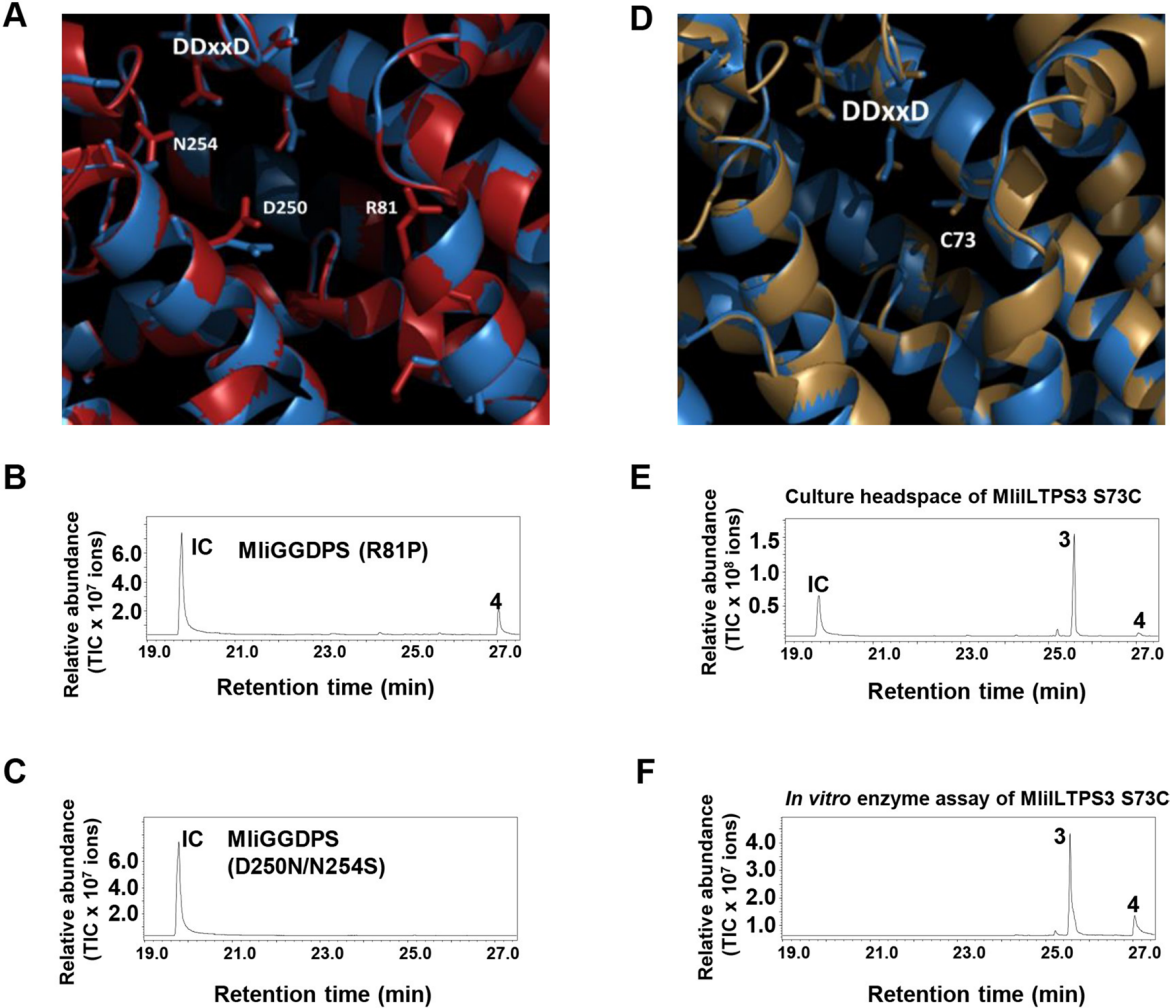
Key amino acids for terpene synthase activities of ILTPSs. (**A**) Homology-based structural model of MliGGDPS (red) and MliILTPS2 (blue). Shown are three amino acids in the active sites that differ between GGDPS and ILTPSs. Numbering of residues according to MliGGDPS. (**B**) Volatiles from the headspace of *E. coli* cultures expression recombinant MliGGDPS R81P. (**C**) Volatiles from the headspace of *E. coli* cultures expression recombinant MliGGDPS D250N/N254S. (**D**) Homology-based structural model of the (*E,E*)-α-farnesene synthase MliILTPS2 (blue) and the (*E*)-nerolidol synthase MliILTPS3 (sand). Shown is one amino acid in the active sites that differ between MliILTPS2 and MliILTPS3. The positon of the amino acid is numbered according to MliILTPS2. (**E**) Volatiles from the headspace of *E. coli* cultures expression recombinant MliILTPS3 S73C. (**F**) In vitro sesquiterpene products formed from recombinant MliILTPS3 S73C using FDP as substrate. 3, (*E,E*)-α-farnesene; 4, (*E*)-nerolidol. *IC* internal compound indole.

High levels of sequence similarities between GGDPSs and ILTPSs (Fig. S1) suggest that their distinct catalytic activities may be determined by a few key amino acids. To test this hypothesis, we first compared the homology-based structure models of MliGGDPS and MliILTPS2. Among the different amino acids, three are located in the active sites: Arginine (R81), aspartic acid (D250), and asparagine (N254) according to the positions in MliGGDPS (Fig. 4A). To test the functionality of these three amino acids, based on sequence comparisons (Fig. S1), one single mutant R81P and one double mutant D250N/N254S was created using MliGGDPS as the wild type enzyme. These two mutant enzymes were first tested for their IDS activity using FDP and IDP as co-substrates. The mutation of arginine 81 to proline in MliGGDPS (MliGGDPS R81P) caused a complete loss of GGDPS activity. In contrast, the D250N/N254S double mutant remained the GGPPS activity (Fig. S8), despite a reduced relative activity (approximately 40% of the activity of the wild type enzyme).

Next, we analyzed MliGGDPS R81P and MliGGDPS D250N/N254S to determine whether they acquire terpene synthase activity using the culture headspace technique. While no sequiterpene was detected from the headspace of *E. coli* culture expressing MliGGDPS D250N/N254S (Fig. 4C), (*E*)-nerolidol was produced by the *E. coli* culture expressing MliGGDPS R81P (Fig. 4B).

There are two types of ILTPSs in both *M. larici-populina* and *M. lini*: (*E*)-nerolidol synthase and (*E,E*)-*α*-farnesene synthase. As noted earlier, these two types of ILTPSs also shared high sequence similarities (Fig. S1). To understand the structural basis underlying their functional divergence, we performed both sequence alignment and homology-based structural modeling. One candidate amino acid for functional divergence is serine (S73) in (*E*)-nerolidol synthase and a cysteine in the equivalent position of (*E,E*)-*α*-farnesene synthase (Figs. 4D, S1). Notably, this residue is in the fourth position upstream of the FARM of IDSs and it has been demonstrated to be critical in determining chain length of prenyl diphosphate products^6,7,30,31^. To test the functionality of this residue, we created a S73C mutant using the (*E*)-nerolidol synthase MliILTPS3 as wild type enzyme. The MliILTPS3 S73C mutant produced mainly (*E,E*)-*α*-farnesene from FDP (Fig. 4E,F), indicating a successful functional switch from (*E*)-nerolidol synthase to (*E,E*)-*α*-farnesene synthase.

### Evolutionary analysis of *ILTPS* genes

**Figure 5:**
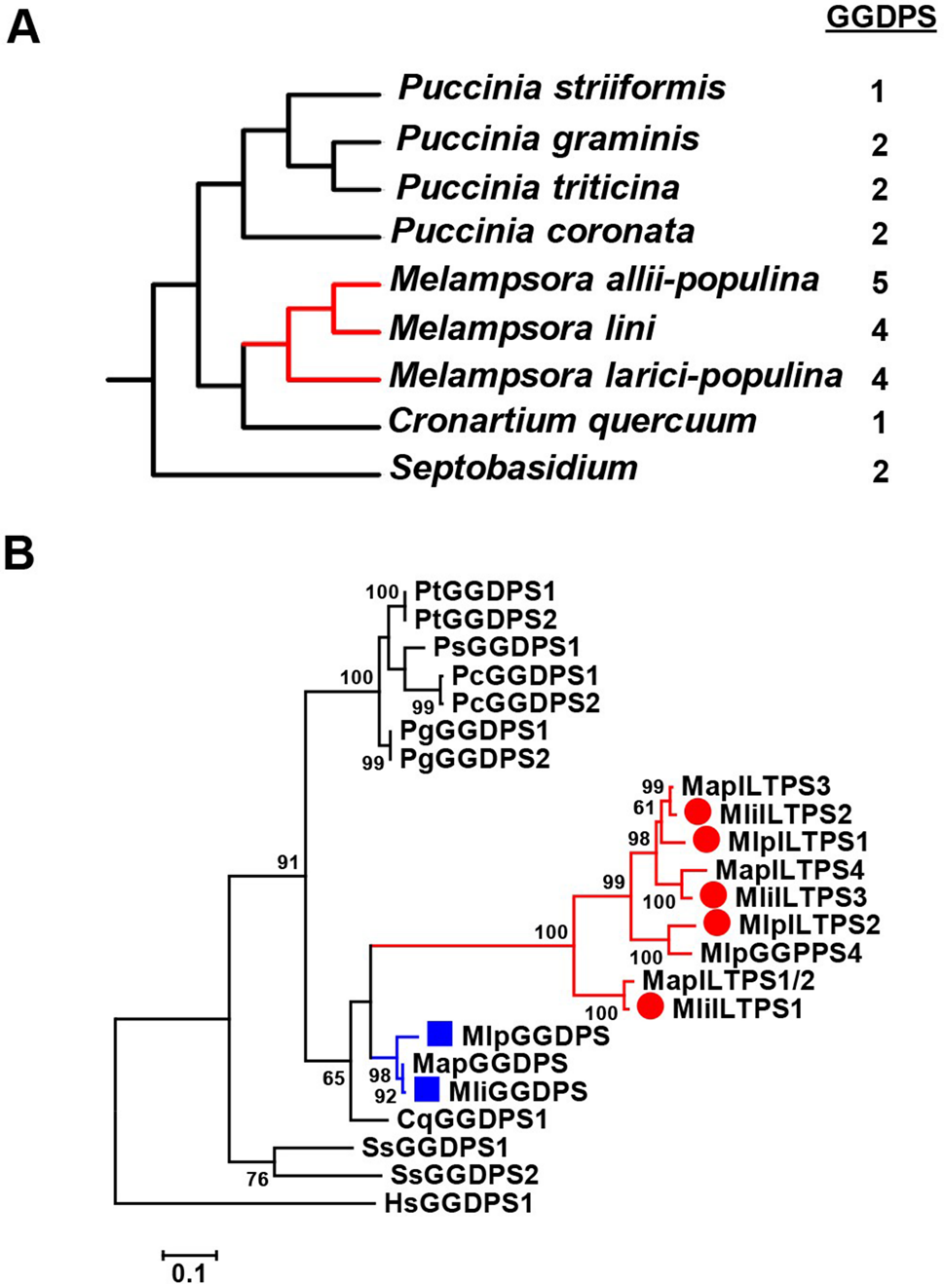
Phylogenetic analysis of the members of the GGDPS family in the class of Pucciniomycetes. (**A**) The number of *GGDPS* genes identified in nine species. The phylogeny was generated according to https://genome.jgi.doe.gov/programs/fungi/index.jsf. (**B**) Phylogenetic analysis of GGDPSs from *Cronartium quercuum* (Cq), *Melampsora allii­-populina* (Map), *Melampsora lini* (Mli), *Melampsora larici-populina* (Mlp), *Puccinia coronata* (Pc), *Puccinia graminis* (Pg), *Puccinia striiformis* (Ps), *Puccinia triticina* (Pt), and S*eptobasidium PNB30-8B* (Ss). The human (*Homo sapiens*) HsGGDPS (NP_001032354.1)^32^ was used as outgroup. Bootstrap values (based on 1,000 replications) greater than 50% are shown for the corresponding nodes.

To understand the evolutionary origin of the newly identified IDS-like *TPS* genes and *GGDPS* genes in fungi, we analyzed the putative *GGDPS* family identified in nine sequenced species of fungi in the class of Pucciniomycetes (https://genome.jgi.doe.gov/portal/), to which *Melampsora* belong (Fig. 5A). First, we analyzed the number of putative *GGDPS* genes in each of the nine species. Four of the nine species contain two putative *GGDPS* genes, and two of them contain a single *GGDPS* gene (Fig. 5A; Table S1). In addition, we searched the genome sequences of the nine fungal species for classic terpene synthase genes, but none was identified. Next, phylogenetic analysis including all the putative *GGDPS* genes from these nine species and the human *GGDPS* gene (outgroup) were performed (Fig. 5B). In this rooted phylogenetic tree, two putative *GGDPS* from *Septobasidium* lied at the base of the tree. The rest of the putative *GGDPSs* form three clades: one clade contained both *bona fide* Mli*GGDPS* and MlpGGDPS characterized in our study, a putative *GGDPS* from *M. allii-populina* and one putative *Cronartium quercuum GGDPS.* The second clade only contained all functional validated IDS-like TPSs from *M. larici-populina* and *M. lini* in this study and putative IDS-like *TPSs* from the third *Melampsora* species (*M. allii-populina*). The last clade consisted of putative GGDPSs from the genus *Puccinia* including *Puccinia coronata*, *Puccinia graminis*, *Puccinia striiformis*, and *Puccinia triticina.*

### Expression analysis of *MlpILTPS* genes in *M. larici-populina*-infected poplar leaves

**Figure 6:**
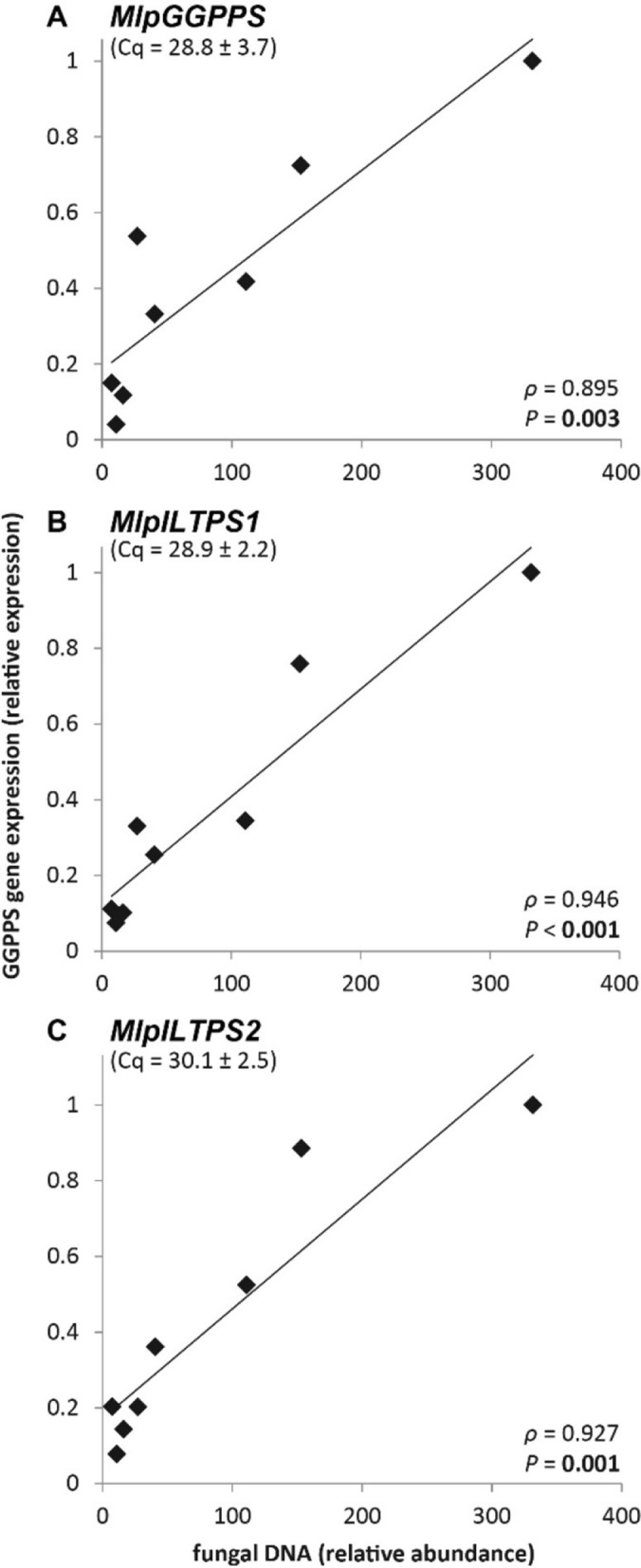
Correlation of fungal abundance (measured as abundance of fungal genomic DNA) and transcript accumulation (relative expression) of (**A**) *MlpGGDPS*, (**B**) *MlpILTPS1*, and (**C**) *MlpILTPS2* in black poplar (*Populus nigra*) leaves infected with *M. larici-populina* 14 dpi (days post infection). The average values of quantitation cycle (Cq) with standard deviation are given for each gene. Pearson correlation (*n* = 8), correlation coefficient *ρ* and significance level *P*.

With *ILTPS* genes in *Melampsora* inferred to have a relatively recent evolutionary origin (Fig. 5), it is intriguing to ask whether these newly evolved genes have acquired biological functions. To address this question, we analyzed the expression of the two functionally characterized *ILTPS* genes *MlpILTPS1* and *MlpILTPS2* in *M. larici-populina*. As a comparison, the expression of *MlpGGDPS* was also measured. As an obligate biotroph, *M. larici-populina* cannot be cultured independently from the host plant. Therefore, we measured the expression of the three genes in poplar leaves at 14 days post-infection by *M. larici-populina*. The transcript abundance of all three genes, *MlpILTPS1*, *MlpILTPS2,* and *MlpGGDPS*, in the poplar leaves correlated strongly with the presence of the fungus itself (Fig. 6), which indicates in vivo expression of the three genes. Moreover, the values of quantitation cycle (Cq) of all three genes were in a similar range (Fig. 6), indicating similar expression levels.

## Discussion

The identification of IDS-like terpene synthase (ILTPS) genes in fungi provides evidence to support our hypothesis that the evolution of terpenes synthases (TPSs) from isoprenyl diphosphate synthase (IDSs) has been a recurrent process. The first occurrence most likely happened at an early stage in the evolution of life as classic TPSs and IDSs do not share significant sequence similarities. In contrast, *ILTPS* genes identified in this study evolved relatively recently. Their origin is not shared by the common ancestor of the class of Pucciniomycetes and may be specific to the genus *Melampsora* (Fig. 5). It is interestingly to note that while ILTPSs from *Melampsora* evolved from geranylgeranyl diphosphate synthase (GGDPS), several IDS-like terpene synthases identified from insects were derived from farnesyl diphosphate synthase (FDPS)^17–19^. This indicates not only an independent occurrence of the evolution of terpene synthases from IDSs in distantly related lineages but also distinct paths in which different types of IDSs served as progenitors. The newly discovered *ILTPS* genes expand the types of terpene-producing genes in fungi, which include classic *TPS* genes, *ubiA* type terpene synthase gene and the *TPS-IDS* fused genes^14^. It is worth noting that classic *TPS* genes are known to exist only in plants^9^, fungi^14^, bacteria^15^, social amoeba^16^, and red algae^33^. Nonetheless, diverse organisms such as the malaria parasite *Plasmodium falciparum*^34^, rats^35^, and elephants^36^, are known to accumulate terpenoids but lack classic *TPS* genes. It will be interesting future research to determine whether terpenoids in such organisms are de novo synthesized, if so, whether by the action of IDS-like terpene synthases or other novel terpene-producing enzymes.

The evolution of *Melampsora ILTPS* genes was driven by gene duplication followed by functional divergence, particularly through neofunctionalization caused by the R81P mutation. Duplication of a *bona fide GGDPS* gene was the beginning of *ILTPS* gene evolution. After duplication, while one copy retained its original GGDPS function, the other copy evolved novel catalytic activity as TPS through the accumulation of mutations. Since its origination, ancestral *ILTPS* gene had further evolved. First, multiple rounds of gene duplication had occurred, resulting in three or more copies of *ILTPS* genes in each *Melampsora* species (Fig. 5). Second, functional divergence of duplicated *ILTPS* genes had occurred, leading to distinct catalytic activities (Fig. 3). By performing homology-based structural modeling, sequence comparisons and site-directed mutagenesis, we were able to identify one critical residue in determining product specificity of ILTPSs (Fig. 4E,F). For many TPSs, a single or a few amino acid changes can lead to novel catalytic activities^37,38^. Our results show that simple changes in product specificities can happen to ILTPSs like in classic TPSs through the changes of a few key amino acids.

What factors drove the loss of coupling activity of an IDS and acquisition of a terpene synthase activity? While our successful identification of fungal *ILTPS* genes in this study relied on the initial comparison of the hallmark metal-binding motifs between IDSs and TPSs (Fig. 1), the change of conserved SARM to “NDxxSxxxE” is not sufficient to make this activity switch based on two pieces of evidence. The first piece of evidence comes from MliILTPS1, which contains a conserved SARM (Table 1) but behaved like other ILTPSs (Figs. 2 and 3), suggesting that the “NSD” triad is not absolutely required for TPS activity. The second piece of evidence comes from site-directed mutagenesis studies. The MliGGDPS D250N/N254S mutant did not show terpene synthase activity (Fig. 4C). For structurally characterized IDSs, the first aspartate of the FARM motif coordinates Mg^2+^_A_ and Mg^2+^_C_, while the first aspartate of the SARM motif coordinates Mg^2+^_B_^13^. In a number of structurally characterized class I TPSs from bacteria^39^ and fungi^40^, which contain one aspartate-rich motif and one “NSD” triad, Mg^2+^_A_ and Mg^2+^_C_ are also coordinated by the first aspartate of the aspartate-rich motif, like IDSs. In contrast, the coordination of Mg^2+^_B_ in TPSs involves three amino acids, asparagine, serine, and glutamate, in the “NDxxSxxxE” motif. Although our results showed that a “NSD” triad is not absolutely required for terpene synthase activity, it will be an interesting future research to characterize the detailed mechanisms underlying the conversion of IDSs, a coupling enzyme for chain elongation, to TPSs, by specifically assessing the role of the changes of SARM to “NSD” triad. Despite the inconclusive role of the second “aspartate-rich” motif, by performing homology-based structural modeling, sequence comparisons, and site-directed mutagenesis, we were able to identify one important residue R81 of MliGGDPS in determining IDS versus TPS activity. We should acknowledge that the mutant had lower TPS activity than ILTPSs (Fig. 4B), suggesting the importance of other amino acids for terpene synthase activity. Nonetheless, the MliGGDPS R81P mutation led to the loss of IDS activity and acquisition of TPS activity (Fig. 4A,B), supporting the importance of this change in the evolution of ILTPSs from GGDPS. In previous structural studies, the arginine in a bacterial FDPS in the equivalent position of R81 was hypothesized to be involved in forming a salt bridge to interact with IDP pyrophosphate^41^. It is also interesting to note that when the “KKxR” motif in MhFDPS, a FDS from the insect *M. histrionica*, were substituted with a SDAW motif found in MhTPS, a FDS-like terpene synthase, the MhFDPS mutant lost the IDS activity^17^. The arginine in “KKxR” of MhFDPS is in the equivalent positon of R81. Taken together, these results support a critical role of R81 in the activity of IDSs and the importance of its mutation leading to ILTPSs. The S73C change in MliILTPS3 led to the change of product from (*E*)-nerolidol to (*E,E*)-*α*-farnesene (Fig. 5F). Interestingly, this same residue in IDSs has been demonstrated to determine chain length of phenyl diphosphate products^6,7,30^. Future structural studies of ILTPSs may provide a mechanistic explanation for the role of this residue in product specificity of ILTPSs.

What are the biological significance of *ILTPS* genes? Being obligate biotrophic plant parasites, *Melampsora* species are challenging to investigate. Nevertheless, the detection of *ILTPS* transcripts in poplar leaves inoculated by *M. larici-populina* (Fig. 6) suggests that these newly evolved terpene synthase genes are involved in the biology of *M. larici-populina*. Recent genomic studies^25^ suggested that one highly advantageous adaptation of obligate, pathogenic fungi including *Melampsora* is that their genomes are configured for rapid evolution^42^. The appearance of *Melampsora*-specific *ILTPS* genes may be the result of such rapid genome evolution. The terpene products of ILTPSs occur in many other organisms such as plants, where they function in plant-environment interactions^2^. In contrast, our understanding of the functions of fungal terpenes is still rather limited. A recent study showed that *P. nigra* leaves infected by *M. larici-populina* emited a number of terpene volatiles, including (*E*)-β-ocimene and (*E*)-4,8-dimethyl-1,3,7-nonatriene (DMNT), in higher amounts than uninfected leaves^43^. (*E*)-β-ocimene is the main monoterpene product of MlpILTPS1 (Fig. 3A). DMNT is known to be produced from (*E*)-nerolidol^44^, which is the main sesquiterpene product of MlpILTPS2 (Fig. 3C). Although speculative, it will be interesting to determine whether IDS-like TPSs contribute to the production and emission of (*E*)-β-ocimene and DMNT from *M. larici-populina*-infected poplar trees, and, if so, how the production of such terpenoids contribute to the lifestyle of *M. larici-populina*. The continual development of a toolbox for *Melampsora* will provide new opportunities for investigating the specific biological function of fungal *ILTPS* genes.

## Experimental

### Chemicals and reagents

The radiolabeled [1-^14^C]-IDP (55 mCi/mmol) and [1-^3^H]-FDP (20 Ci/mmol) were purchased from American Radiolabeled Chemicals (St. Louis, MO). (*E*)-GDP, (*E*,*E*)-FDP, and (*E*,*E*,*E*)-GGDP were obtained from Echelon Biosciences (Salt Lake City).

### Sequence retrieval and analysis

A standard BLASTP search against the NCBI (https://www.ncbi.nlm.nih.gov/) non-redundant protein sequences from fungi (taxid:4751) was performed using four queries: a farnesyl pyrophosphate synthase (Q92250 from *Neurospora crassa*), a geranylgeranyl diphosphate synthase (Q12051 from *Saccharomyces cerevisiae*), a geranylgeranyl diphosphate synthase (Q9RUJ1 from *Deinococcus radiodurans*), and a solanesyl diphosphate synthase (XP_023471551 from *Rhizopus microsporus*). All putative fungal *trans*-IDSs identified through the BLASTP search were further examined using HMM search^45,46^. Only the ones containing a polyprenyl synthases domain (PF00348) and in the length of 200–500 amino acids were considered verified *trans*-IDSs. An inhouse script was used to analyze SARM. Multiple sequence alignments were performed using MAFFT (version 7.369b, under L-INS-I strategy)^47^. Approximately-maximum-likelihood phylogentic trees were constructed using FastTree (version 2.1.10, under the JTT + CAT model with 1,000 resamples)^48^.

### Gene cloning, protein expression and purification of recombinant enzymes

Total RNA from the fungus *M. larici-populina* was isolated together with the host RNA from the leaf tissue of black poplar (*Populus nigra*) trees. The treatment of trees, RNA isolation and cDNA synthesis were conducted as previously reported^43^. The synthesized first-strand cDNAs were used as template for PCR with the four pairs of primers corresponding to the beginning and the end of coding sequences for MlpGGDPS and MlpILTPSs (Table S3). PCR products were cloned into the pET-32a vector and fully sequenced. For the four genes from *M. lini*, a full-length cDNA for each gene was synthesized by GenScript (https://www.genscript.com/) and cloned to pET-32a vector. All plasmids were transformed into *E. coli* strain BL21 (DE3) pLysS (Life Technologies) for recombinant protein production. Recombinant proteins with a N-terminal His-tag were purified using His-select nickel affinity gel (Sigma). Purity and concentrations of individual recombinant proteins were measured using SDS-PAGE.

### IDS enzyme activity assays

Two protocols were employed for IDS enzyme assays. The purpose of the first protocol was to determine specific prenyl diphosphate products. In this protocol, each assay of 100 µL containing 25 mM MOPS (pH 7.0), 2 mM dithiothreitol, 10 mM MgCl_2_, 1 µL 4 mM various substrate (DMADP, GDP, or FDP), 0.5 µL IDP, 0.5 µL of [1-^14^C]-IDP (American Radiolabeled Chemicals) and 5 µg of purified recombinant protein was incubated at 30 °C for 60 min. The reactions were terminated by adding 1 µL bovine intestine alkaline phosphatase (New England Biolabs) and 1 µL potato apyrase (Sigma-Aldrich) with 0.5 mL of hexane overlaid for overnight. Then the hexane phase were vortexed and concentrated to 20 µL with nitrogen gas. Finally, the prenyl alcohols were separated using a reverse-phase (C18 silica gel-60 matrix, F254S) TLC plate (Merck). TLC plates were measured by Quantity One software. The products of a functional heteromeric geranylgeranyl diphosphate synthase, composed of an enzymatically active large subunit (CsaLSU) and an inactive small subunit (type I) (CsaSSU I) of cucumber (*Cucumis sativus*), synthesizing C_10_-GDP and C_20_-GGDP, were used as a positive control^27^.

The purpose of the second protocol for IDS enzyme assays was to measure the relative specific activity of the tested enzymes using FDP as substrate. The setup of enzyme reactions was the same as that for the first protocol, except that 400 µL of hexane was added to overlay the reaction mixtures. After incubating at 30 °C for 30 min, the reaction was terminated by adding 20 µL of 3 N HCl for acid hydrolysis. After hydrolysis, 200 μL of the hexane phase was extracted and the radioactivity was measured using a scintillation counter (LS6500 Beckman).

### TPS enzyme activity assays and kinetic measurements

Two protocols were employed for TPS enzyme assays. The first protocol was an in vitro method. In this protocol, reaction mixtures containing various substrates (GDP, FDP, or GGDP) were incubated at room temperature for 60 min. The volatile products captured by solid phase microextraction (SPME) fiber were analyzed by GC–MS (Shimadzu GC 17A). The second protocol was an in vivo method that relies on the internal pool of FDP of *E. coli* for the measurement of sesquiterpene synthase activities, which was recently described^28^. When OD_600_ of *E. coli* cultures (50 mL) reached 0.5, isopropyl-β-d-thiogalactoside was added to the culture at a concentration of 0.5 mM to induce expression of ILTPSs. After induction at room temperature (22 °C) overnight, SPME was used to collect volatile compounds from the headspace of each culture for one hour and then inserted into GC for separation and identification as described for in vitro enzyme assays. GGDPSs were also assayed to validate the effectiveness of protocol and used as a negative control for sesquiterpene synthase. The identities of the terpene products were determined by comparing their retention times and mass spectra of those in the NIST11, WILEY8, and Adams4 databases and those of authentic compounds (nerolidol, linalool). Nerolidol was kindly provided by Wilhelm Boland (Max Planck Institute for Chemical Ecology) and linalool was purchased from Carl Roth GmbH (https://www.carlroth.com). Kovats retention indices were calculated following the protocol described by Girard^49^.

Kinetic properties of a selected ILTPS using FDP as substrate were determined using a radiochemical approach as previously described^50^. Individual recombinant ILTPSs expressed in *E. coli* were purified and used for kinetic measurements using [1-^3^H]-(*E*,*E*)-FDP as substrate. Each reaction assay of 50 µL containing 10 mM MOPSO, pH 7.0, 10 mM MgCl_2_, 0.2 mM NaWO_4_, 0.1 mM NaF, 0.05 mM MnCl_2_, 1 mM DTT, 10% glycerol (v/v), and 0.5 µg of purified recombinant protein was incubated at room temperature overlaid with 150 µL of hexane. After 10 min, the reaction mixture was vortexed and 75 μL from the organic phase was removed to measure radioactivity using a scintillation counter. Three independent assays were performed and the apparent *K*_m_ and *k*_cat_ values were calculated using the Hyperbolic Regression Analysis (HYPER 1.01) software (J.S. Easterby, University of Liverpool).

### Homology-based structural modeling and site-directed mutagenesis

Homology-based structure models of MliGGDPS, MliILTPS2, and MliILTPS3 were created using the SwissModel web service (swissmodel.expasy.org) and the crystal structure of human GGDPS Y246D (6c56.1.A) as template. Visualization of the models was done using PyMOL (https://pymol.org/). The QuickChange site-directed mutagenesis kit (Agilent, Santa Clara, USA) was used for generating various mutants. Primers were designed according to the manufacturer’s instructions and listed in Table S3.

### Quantification of fungal abundance and transcript levels in infected poplar leaves

The general procedure for infection of black poplar (*P. nigra*) trees with *M. larici-populina*, RNA isolation, cDNA synthesis, and quantification of fungal abundance was performed as previously reported^43^. In short, young black poplar trees (eight different genotypes = eight replicates) were spray-inoculated with uredospores of *M. larici-populina* and leaves were flash-frozen in liquid nitrogen 14 days post-infection. Genomic DNA was used to quantify fungal abundance by qRT-PCR using primers specific to the ITS region of *M. larici-populina*. cDNA was used to quantify transcripts of the *MlpGGDPS* and *MlpILTPSs* genes from *M. larici-populina* using specific primers (Table S3). The qRT-PCR for all genes was performed in technical duplicates on a CFX Connect Real-Time PCR Detection System (Bio-Rad) using the following parameters: 95 °C (3 min), 40 cycles of 95 °C (30 s) + 60 °C (30 s), melt curve from 53 to 95 °C. Data were normalized to poplar *Actin 2*^51^. The reaction mixture contained Brilliant III Ultra-Fast SYBR Green QPCR Master Mix (Agilent), 1 µL of DNA or cDNA, and 10 µmol of forward and reverse primers. Correlation between fungal abundance and transcript abundance was evaluated using SPSS (SPSS Statistics 17.0).

## Supplementary information

Supplementary Information.

## Data Availability

Data deposition: the sequences reported in this paper have been deposited in the GenBank database (accession nos. MK946437-MK946443).
