# Supplementary material for: Evolution of isoprenyl diphosphate synthase-like terpene synthases in fungi
Source: Sci Rep. 2020 Sep 10;10:14944. doi: 10.1038/s41598-020-71219-z (PMC7484799; doi:10.1038/s41598-020-71219-z)
Supplement: Supplementary file 1 — Supplementary Information. [file 41598_2020_71219_MOESM1_ESM.pdf]

Supplementary Information:

Evolution of isoprenyl diphosphate synthase-like terpene synthases in fungi

Guo Wei, Franziska Eberl, Xinlu Chen, Chi Zhang, Sybille B. Unsicker, Tobias G. Köllner, Jonathan Gershenzon and Feng Chen

Table S1. GGDPSs identified in 6 species the class of Pucciniomycetes

Species	Gene ID	Gene name
Cronartium quercuum	fgenesh1_pm.202_#_11	CqGGDPS1
Puccinia coronata	PCA_SD_04467-T1	PcGGDPS1
	PCA_SD_09689-T1	PcGGDPS2
Puccinia graminis	PGTG_12321T0	PgGGDPS1
	PGTG_19831T0	PgGGDPS2
Puccinia striiformis	maker-PST130_10993-snap-gene-0.3-mRNA-1	PsGGDPS1
Puccinia triticina	PTTG_02381T0	PtGGDPS1
	PTTG_02381T1	PtGGDPS2
Septobasidium PNB30-8B	estExt_fgenesh1_pm.C_60078	SsGGDPS1
	estExt_fgenesh1_pg.C_50169	SsGGDPS2

Table S2. Kovats retention index of (*E*)-β-ocimene and (*E,E*)-α-farnesene.

Identified terpenes	Retention indices[1]	Reference retention indices[2]
(*E*)-β-ocimene	1050.3	1050
(*E,E*)-α-farnesene	1504.9	1505

[1]Retention indices relative to C_7-C_{40} saturated alkanes in this study.

[2]Reference retention indices were obtained by Sparkman.

Sparkman, O. D. Identification of essential oil components by gas chromatography/mass spectroscopy Robert P. Adams. *J Am Soc Mass Spectr* **8**, 671-672, doi:10.1021/jasms.8b01049 (1997).

Table S3. Primers used in this study.

Primer name	Sequence (5' to 3')
**For gene cloning and expression (pET-32a vector)**	
MliGP for	GGATCCATGAAACCTTTACCTTCAACAAATGG
MliGP rev	AAGCTTTCAATGTTGATCTGTTGCAGGTGG
MliILTPS1 for	GAATTCATGACTCTCAGTATCTATGACGACA
MliILTPS1 rev	CTCGAGCTAGCTTTTGTATTTTTCTTCAATA
MliILTPS2 for	GAATTCATGAGTATCTATGATGACATCTTCAA
MliILTPS2 rev	CTCGAGCTATCGGTCTGCGATCTCAGGAATT
MliILTPS3 for	GGATCCATGAGTATCTATGATGACATTTTCA
MliILTPS3 rev	AAGCTTCTATCGGTCTTCAATCTCAGGAGTT
MlpGP for	GGATCCATGAAACCTGTACCTTCAACAAATGG
MlpGP rev	AAGCTTTCAATTTTGATCTGTTGCAGGTGG
MlpILTPS1 for	GGATCCATGAGTATCTATGATGACATTTTG
MlpILTPS1 rev	AAGCTTCTAATGGTCTTCAATCTCAGGAG
MlpILTPS2 for	GGATCCATGATGCTCCCTACTATTGTTG
MlpILTPS2 rev	AAGCTTCTATTGGTCATCAATTTCAGG
**For RT-qPCR**	
qMlpGP for	CAACAACAAAACCGGGGGAC
qMlpGP rev	TCAGGGGTCTCAGGTATCGG
qMlpILTPS1 for	ACCATAAACACAGCCACTTACG
qMlpILTPS1 rev	GTGCCGCAATCTCATCGTTG
qMlpILTPS2 for	ACAAACACCTCCCTACTATTGTTG
qMlpILTPS2 rev	ATGATTGAAAGCCCTGGTGAC
**For site-directed mutagenesis**	
R81P for	CCCTGGGAAAGAAATCCCCTCGATGATGATCGATG
R81P rev	CATCGATCATCATCGAGGGGATTTCTTTCCCAGGG
250254 for	CATCTTTCAAATCAGAAACGATCTCTTGAGCCTTTCCTCTGTTTACAC
250254 rev	GTGTAAACAGAGGAAAGGCTCAAGAGATCGTTTCTGATTTGAAAGATG
S73C for	GGACCAATACATGCTGCCTGTCTATTACTCGACGACATC
S73C rev	GATGTCGTCGAGTAATAGACAGGCAGCATGTATTGGTCC

"for" and "rev" stand for forward primer and reverse primer, respectively.

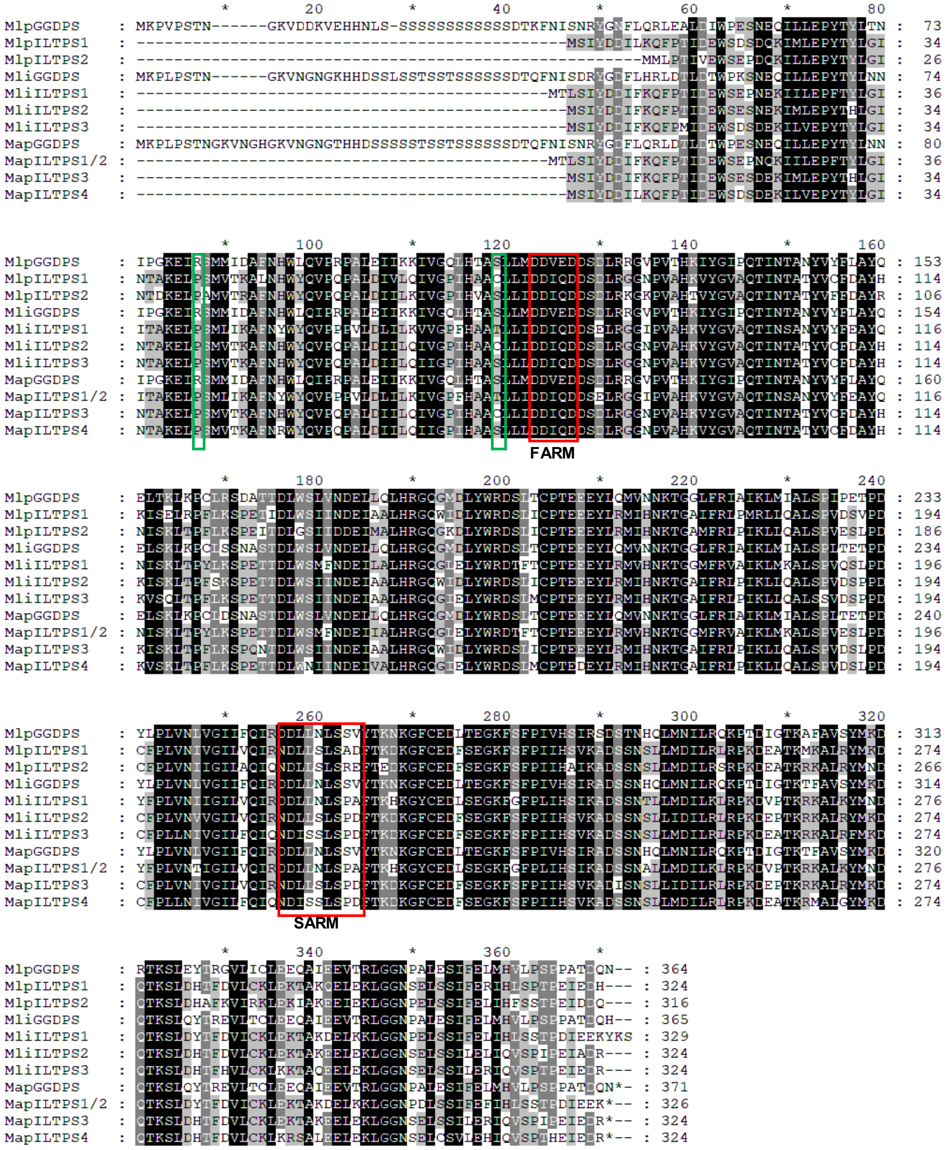

Fig. S1. Multiple sequence alignment of *Melampsora* GGDPS proteins. Each GGDPSs sequences from *Melampsora larici-populina*, *Melampsora lini, and Melampsora allii-populina* were selected. The first highly conserved domains ‘$DD_{XX}D$’(FARM), and second less conserved motif “NSE/DTE” (SARM) were indicated in red boxes. Green boxes mark sequences were selected for site directed mutagenesis. Mlp, *Melampsora larici-populina*; Mli: *Melampsora lini*; Map, *Melampsora allii-populina*.

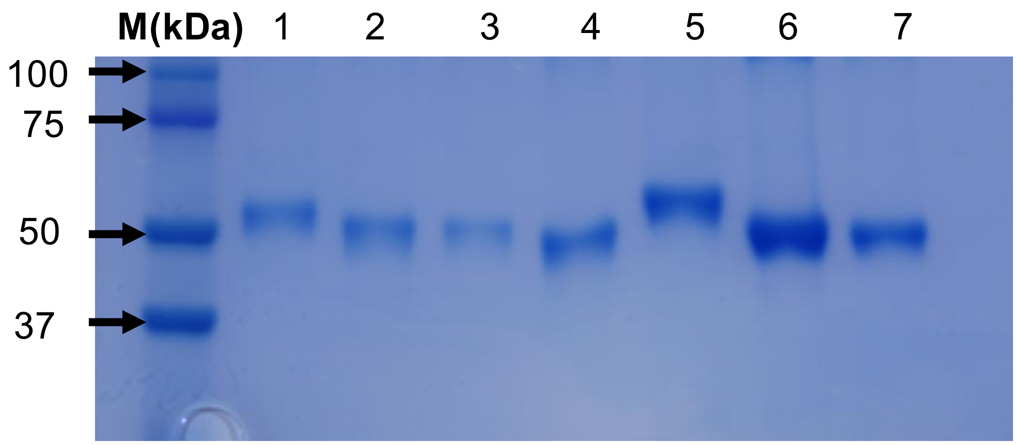

Fig. S2. SDS-PAGE analysis of purified His-tagged proteins. 1: MliGGPPS; 2: MliILTPS1; 3: MliILTPS2; 4: MliILTPS3; 5: MlpGGPPS; 6: MlpILTPS1; 7: MlpILTPS2.

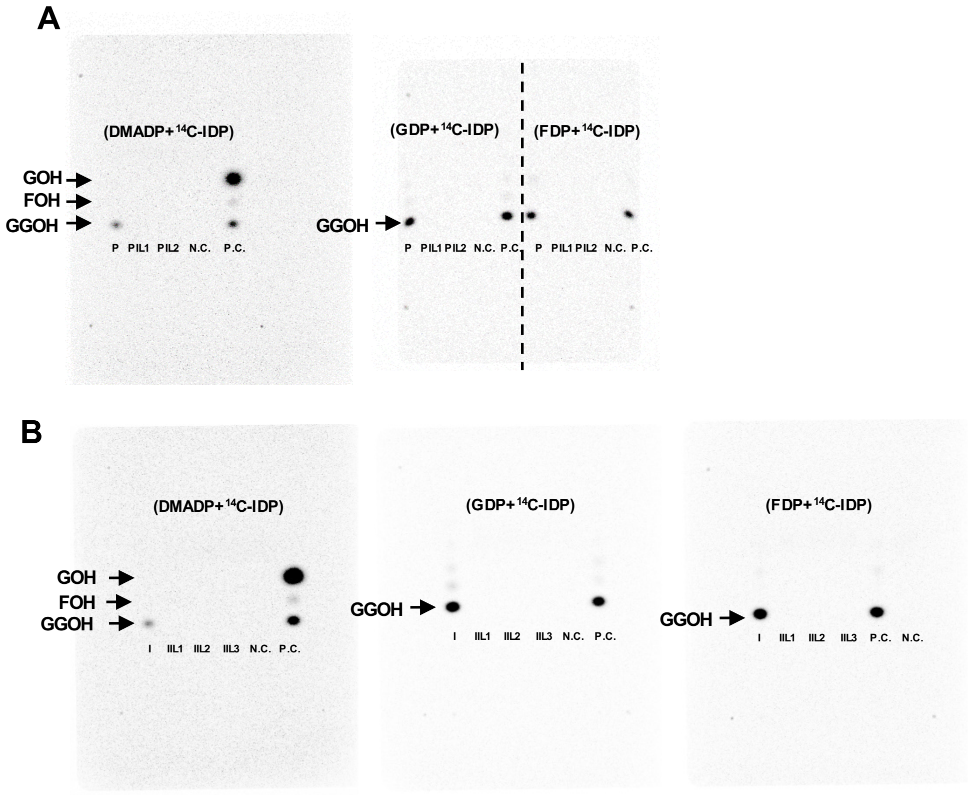

Fig. S3. The original full-length blots presented in Figure 2. All descriptions are the same as in Figure 2. *In vitro* IDS enzymatic assays of the GGDPSs and ILTPSs. (A) Assays of MlpGGDPS (P), MlpILTPS1 (PIL1), MlpILTPS2 (PIL2) from *Melampsora larici-populina* using DMADP, GDP, and FDP together with [1-^{14}C]-IDP as substrates. (B) Assays of MliGGDPS (I), MliILTPS1 (IIL1), MliILTPS2 (IIL2) and MliILTPS3 (IIL3) from *Melampsora lini* using DMADP, GDP, and FDP together with [1-^{14}C]-IDP as substrates.

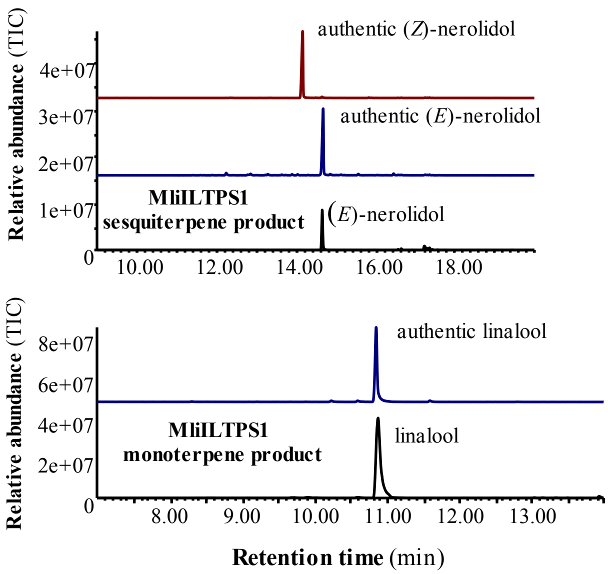

Fig S4. Identification of monoterpene and sesquiterpene products of MliILTPS1. Enzyme products were extracted from the assays with hexane and analyzed using GC-MS. Authentic standards diluted in hexane were used for identification.

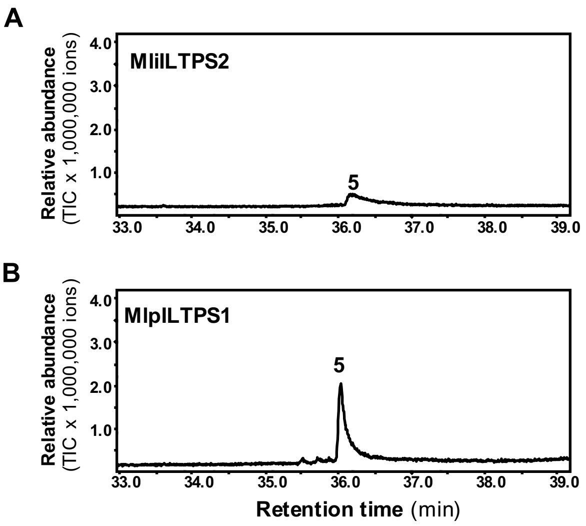

Fig. S5. GC chromatograms of the diterpene products of MliILTPS2 (A) and MlpILTPS1 (B). Recombinant proteins were incubated with GGDP as substrate and reaction products were extracted with hexane and analyzed using GC-MS. The total ion chromatograms (TICs) are shown. 5, (*E,E,E*)-α-springene.

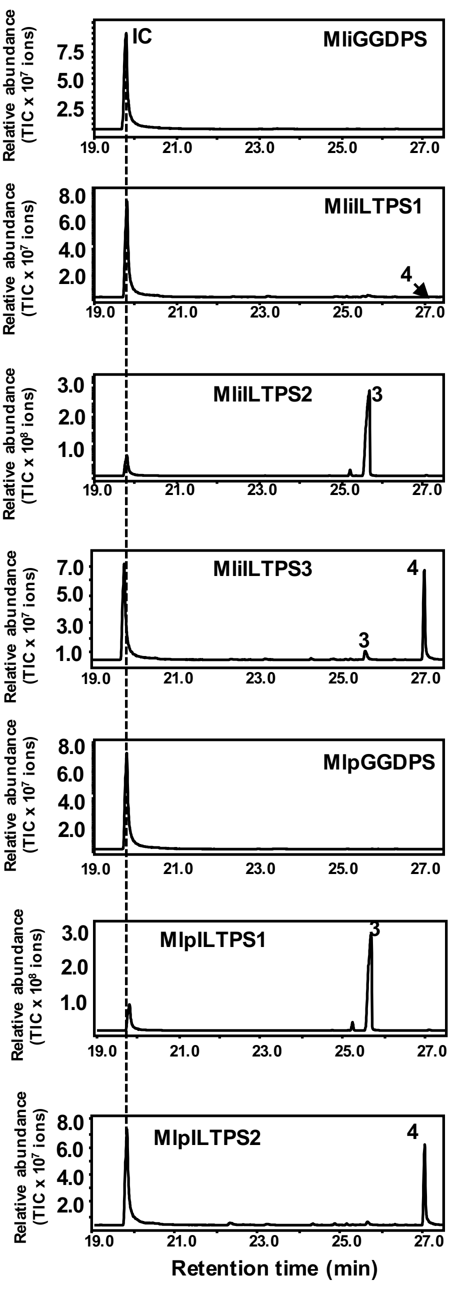

Fig. S6. Sesquiterpene volatile profiles of *E. coli* Bl21-DE3-Star expressing different recombinant enzymes. Volatiles were collected from the headspace of the induced bacterial cultures using SPME and analyzed using GC-MS. 3. (*E,E*)-α-farnesene; 4, (*E*)-nerolidol. "IC" stands for an internal compound indole.

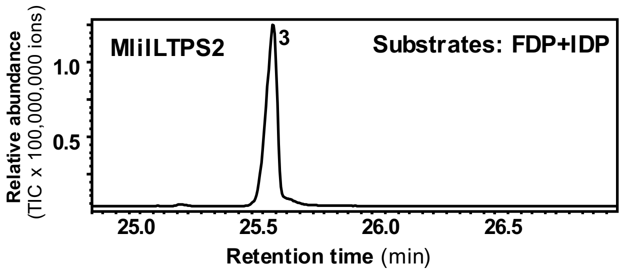

Fig. S7. GC chromatogram of sesquiterpene product of MliILTPS2 using FDP in the presence of IDP. Reaction products were captured and analyzed using GC-MS. The total ion chromatograms (TICs) are shown. 3, (*E*,*E*)-α-farnesene.

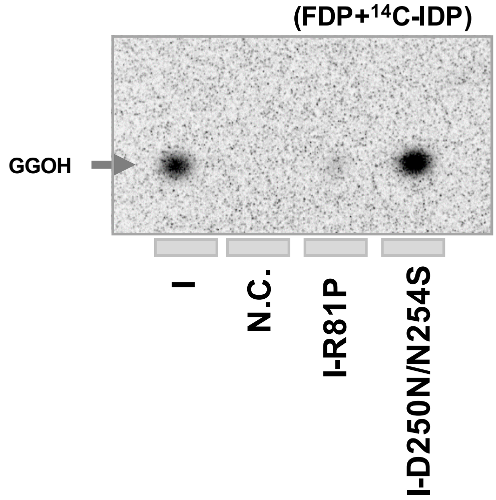

Fig. S8. *In vitro* IDS enzymatic assays of the MliGGDPS and two mutants. Assays of MliGGDPS (I), boiled MliGGDPS (N.C.), MliGGDPS R81P (I-R81P) and MliGGDPS D250N/N254S (I-D250N/N254S) from *Melampsora lini* using and FDP together with [1-^{14}C]-IDP as substrates. The reaction products were separated via thin-layer chromatography (TLC). GGOH, geranylgeraniol.
